# Supplementary material for: SOX2 and SOX9 Expression in Developing Postnatal Opossum (Monodelphis domestica) Cortex
Source: Biomolecules. 2024 Jan 5;14(1):70. doi: 10.3390/biom14010070 (PMC10813269; doi:10.3390/biom14010070)
Supplement: Supplementary file 1 [file biomolecules-14-00070-s001.zip › biomolecules-2765055-supplementary.pdf]

Supplementary information

SOX2 and SOX9 expression in developing postnatal opossum (*Monodelphis domestica*) cortex

Zrinko Baričević [1,†], Marta Pongrac [1,†], Matea Ivaničić [1,‡], Helena Hreščak [1], Ivana Tomljanović [1], Antonela Petrović [1,§], Dan Cojoc [2], Miranda Mladinic [1] and Jelena Ban [1,*]

[1] Faculty of Biotechnology and Drug Development, University of Rijeka, Radmile Matejčić 2, 51000 Rijeka, Croatia; zrinko.baricevic@uniri.hr; marta.pongrac@gmail.com; matea.ivanicic@bi.mpg.de; helena.kusturin4@gmail.com; ivana.tomljanovic00@gmail.com; petrovic.antonela@gmail.com; mirandamp@uniri.hr

[2] CNR-IOM, Materials Foundry, National Research Council of Italy, 34149 Trieste, Italy; cojoc@iom.cnr.it

* Correspondence: jelena.ban@biotech.uniri.hr; Tel.: +385 51 584 576

† These authors contributed equally to this work.

‡ current address: Tools for Bio-Imaging, Max Planck Institute for Biological Intelligence, Am Klopferspitz 18, D-82152 Martinsried, Germany.

§ current address: DMG Research Center, Department of Oncology, University Children's Hospital, University of Zurich, 8001 Zurich, Switzerland.

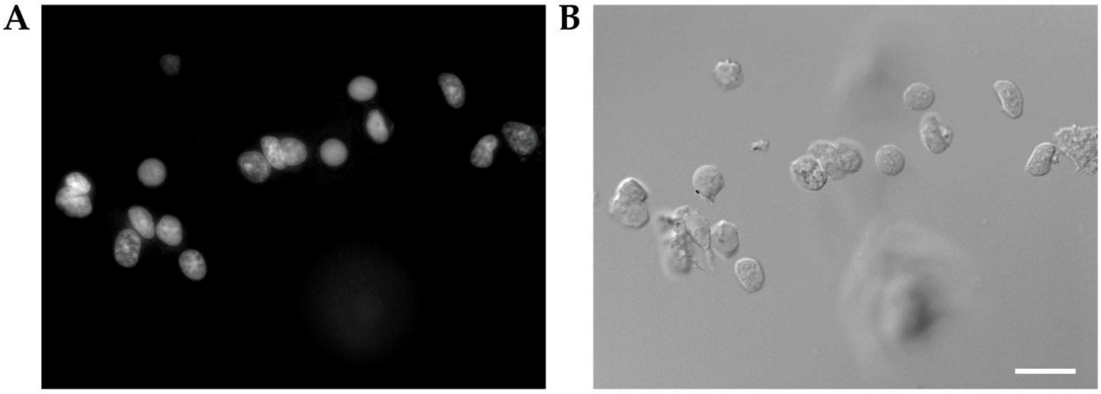

Figure S1. P6 cortex of *Monodelphis domestica* processed with isotropic fractionator method, mounted on a glass slide with coverslip. (A) Cell nuclei stained with Hoechst 33342, projection of 10 µm z-stack acquired with 0.25 µm steps displayed in grayscale. (B) Differential interference contrast (DIC) image of the same optical field confirms that all nuclei are efficiently stained following tissue fixation, homogenization and immunostaining. Images were acquired with 60x 1.42 NA oil immersion objective using Olympus IX83 fluorescent microscope equipped with DIC and fluorescence optics (see Methods). Scale bar, 20 µm.

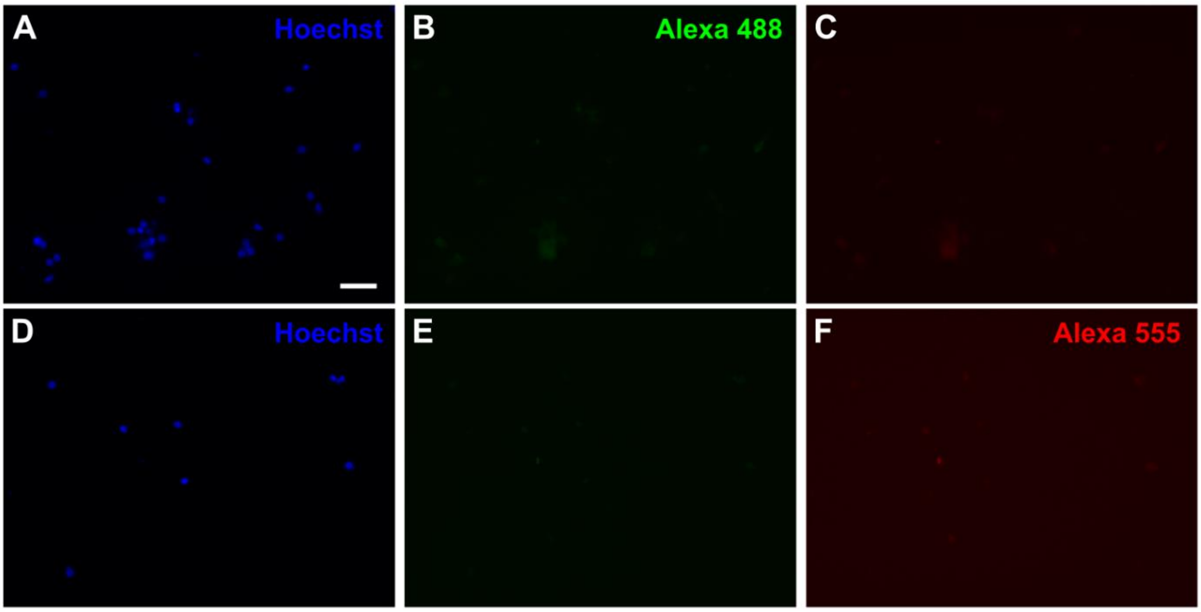

Figure S2. Isotropic fractionator control experiment with omitted primary antibodies. Evaluation of background staining of the secondary antibodies was performed as follows: P30 opossums were processed with isotropic fractionator method as described in Methods and instead of primary antibody, 1% (w/vol) BSA in PBS was used for the overnight incubation. The secondary antibodies goat anti-rabbit Alexa Fluor™ 488 (A-C) and goat anti-rabbit Alexa Fluor™ 555 (D-F) were used separately at the same concentration and incubation time. The same exposure time was kept during imaging. 20x and 0.5 NA objective was used. For every experiment three channels (Olympus fluorescence filter cubes U-FUNA, U-FBW and U-FGW, respectively) were acquired. Hoechst 33342-labelled nuclei (A and C) served as a reference for fluorescence signal of stained sample in both experiments. (A-C) projection of 28 stacks, (D-F) projection of 23 stacks with 1 μm step. Scale bar, 20 μm.

Table S1. Age, body weight, size and number of opossums at postnatal ages from P4 to P30, used in this work.

Age	Body weight (g)	Body size (mm)	Pups used
P4	0.19 ± 0.02	11.5 ± 1.73	11
P5	0.22 ± 0.02	12.6 ± 0.42	12
P6	0.25 ± 0.03	13.5 ± 0.71	8
P16	1.43 ± 0.04	31.20 ± 2.05	3
P17	1.63 ± 0.05	33.33 ± 1.15	6
P18	1.71 ± 0.11	33.77 ± 0.94	7
P30	5.02 ± 0.13	47.75 ± 3.49	8

Table S2. Absolute (total) number of cells in opossum cortex at different ages. A fraction of Hoechst 33342-stained homogenized nuclei of fixed cortices was loaded on hemocytometer and counted using Olympus IX73 inverted fluorescence microscope equipped with long working distance objective (20x, 0.45 NA), fluorescence optics and Olympus XM-10 CCD camera. Isotropic fractionator method was performed as described in Methods.

Age	Total cell number (in millions)
P4-6	5.20 ± 0.31
P16-18	12.72 ± 1.19
P30	21.80 ± 1.00

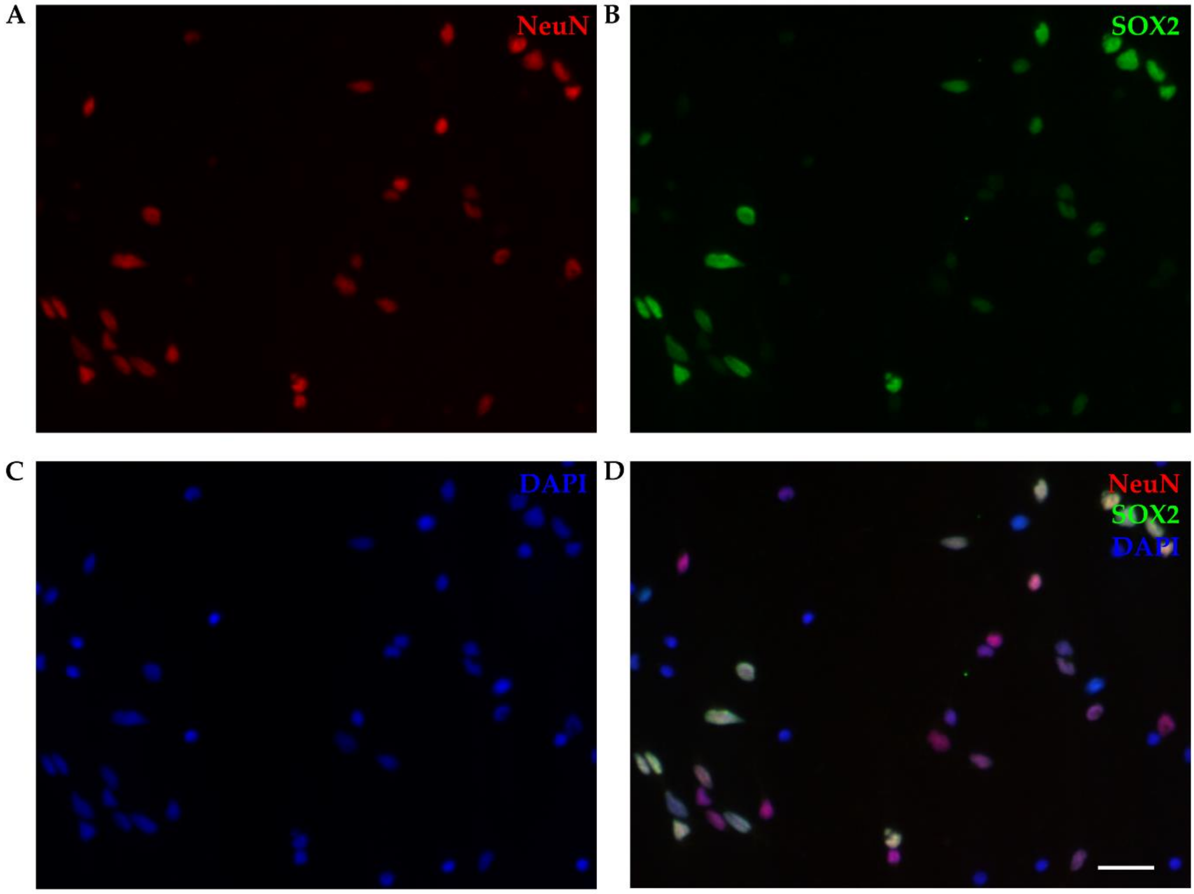

Figure S3. Primary cultures of P4-6 opossum cortex at DIV1. Cells were fixed 24 h after plating and stained for (A) NeuN (red), (B) SOX2 (green), (C) DAPI nuclear stain (blue) and (D) merged. Images are projection of 15 µm stack acquired with 1.27 µm steps using 20x and 0.5 NA objective. Scale bar, 25 µm.

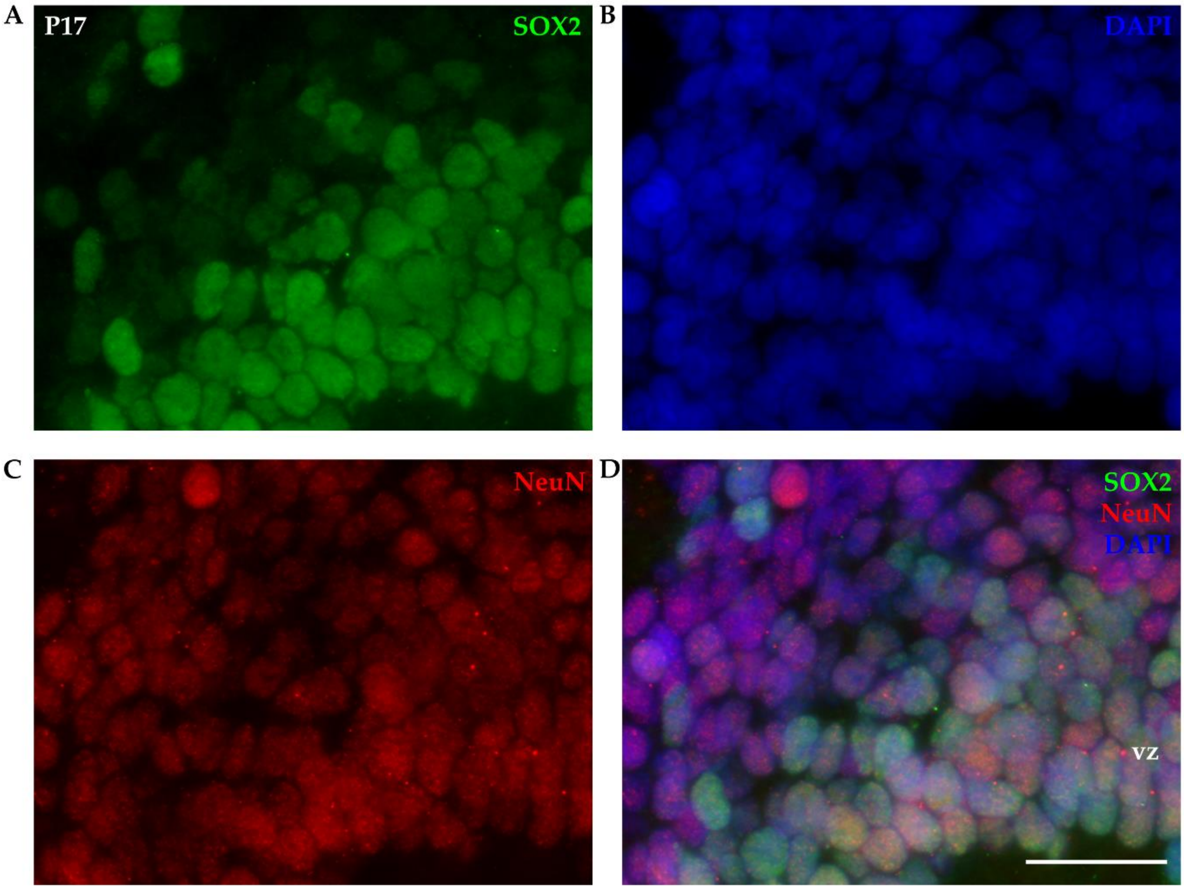

Figure S4. IHC of developing opossum cortex. Coronal sections from P17 opossum cortex were immunostained for (A) SOX2 (green), (B) DAPI (blue), (C) NeuN (red) and (D) merged. Images are projections of 6 µm z-stacks acquired with 0,25 µm steps using 40x 1.4 NA oil immersion objective. vz, ventricular zone. Scale bar, 25 µm.

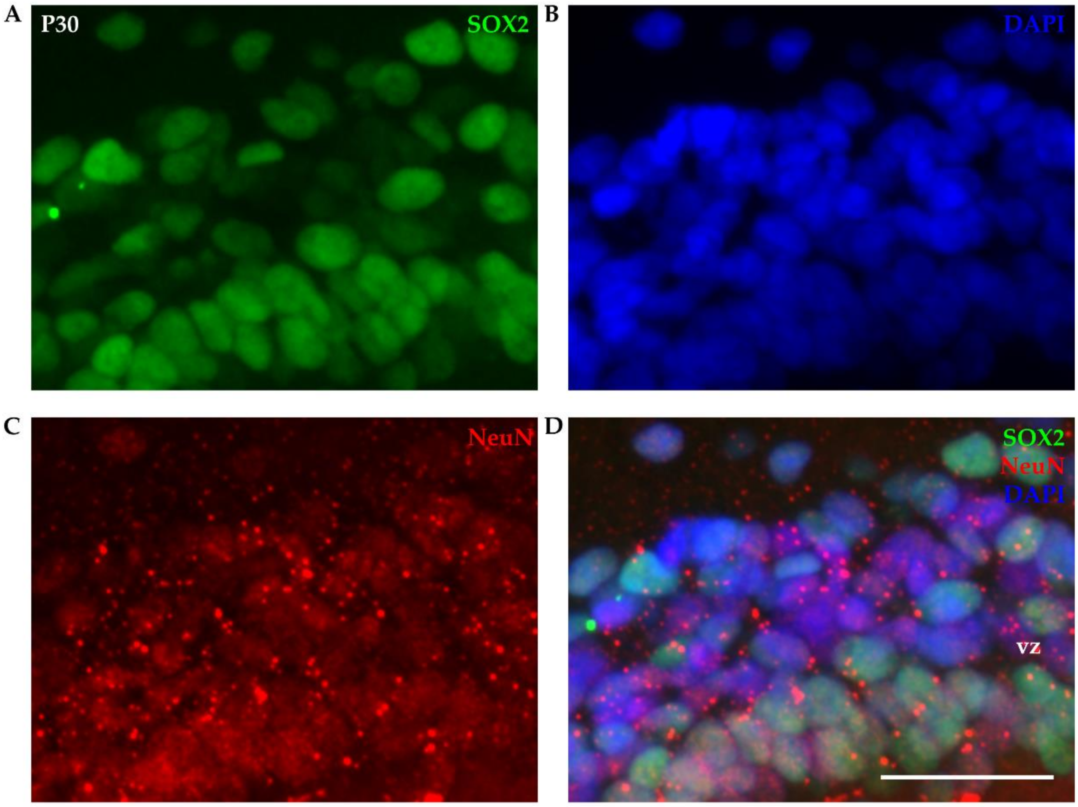

Figure S5. IHC of P30 opossum cortex. Coronal cortical sections were immunostained for (A) SOX2 (green), (B) DAPI (blue), (C) NeuN (red) and (D) merged. Images are projections of 9 µm z-stacks acquired with 0,25 µm steps using 40x 1.4 NA oil immersion objective. vz, ventricular zone. Scale bar, 25 µm.

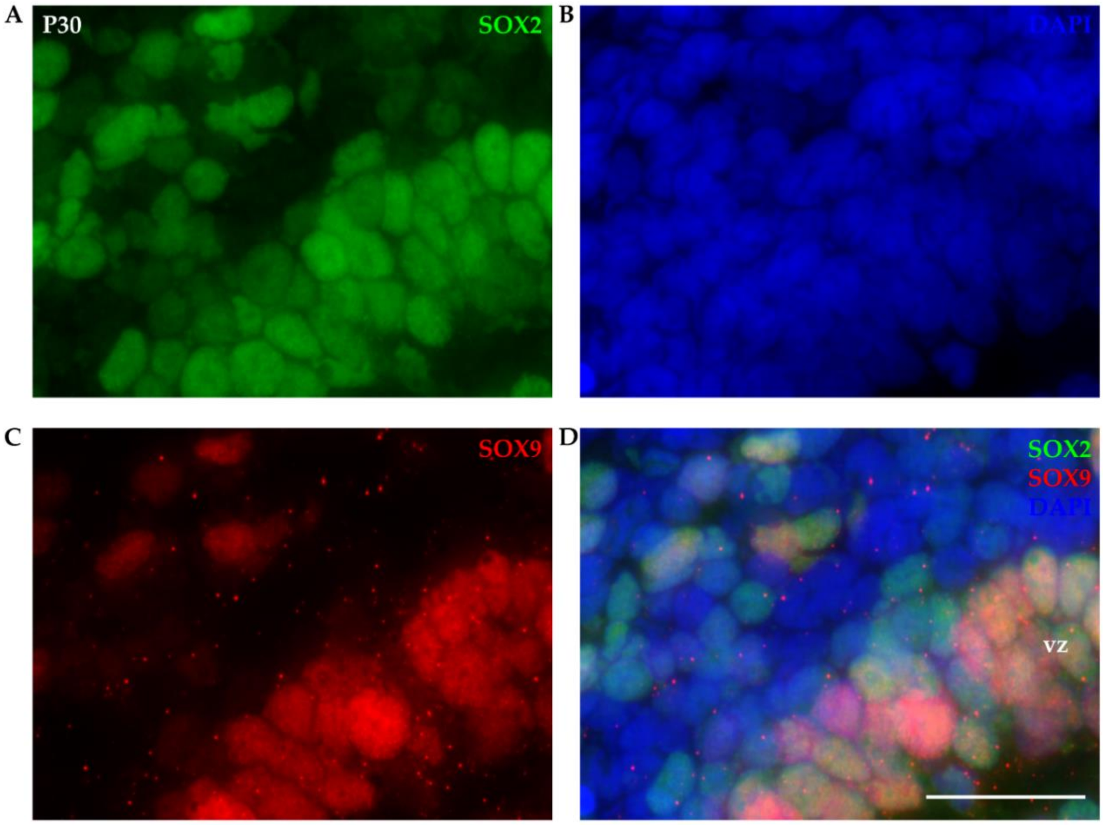

Figure S6. Coexpression of SOX2 and SOX9 during postnatal opossum cortex development analyzed by IHC. Coronal sections from P30 opossum cortex were immunostained for (A) SOX2 (green), (B) DAPI (blue), (C) SOX9 (red) and (D) merged. Images are projections of 10 µm z-stacks acquired with 0,25 µm steps using 40x 1.4 NA oil immersion objective. vz, ventricular zone. Scale bar, 25 µm.

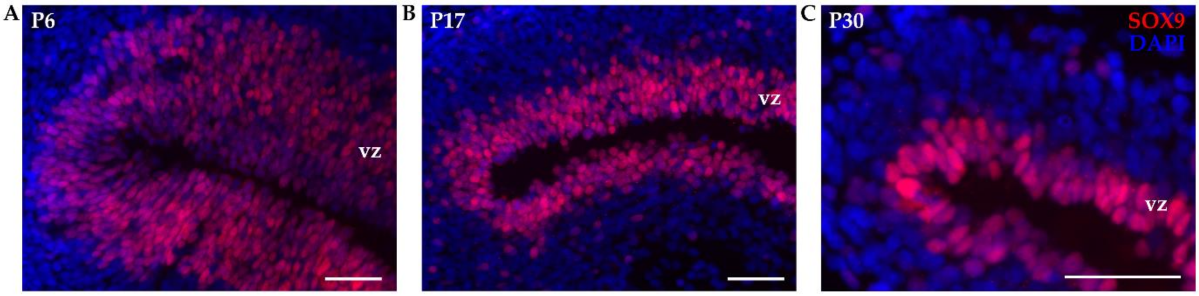

Figure S7. SOX9 expression in ventricular region of developing opossum cortex analyzed by IHC. Coronal sections of (A) P6, (B) P17 and (C) P30 *M. domestica* cortices were immunostained for SOX9 (red) and and cell nuclei were stained with DAPI (blue). Images are projections of 12.5 µm z-stacks acquired with 0,5 µm steps using 20x and 0.5 NA objective. vz, ventricular zone. Scale bar, 50 µm.
